# Impact of body mass index as a continuous variable on short‐ and long‐term outcomes in patients undergoing laparoscopic surgery for colon cancer

**DOI:** 10.1002/ags3.12916

**Published:** 2025-01-23

**Authors:** Takayuki Aiba, Tomonori Akagi, Hidefumi Shiroshita, Kentaro Nakajima, Tetsuji Ohyama, Tatsuya Kinjo, Akiyoshi Kanazawa, Nobuaki Suzuki, Takuya Tokunaga, Manabu Yamamoto, Nobuki Ichikawa, Shungo Endo, Yutaka Kojima, Takatoshi Nakamura, Shuji Saito, Yoshinori Kagawa, Shinobu Ohnuma, Seiichiro Yamamoto, Takeshi Naitoh, Masafumi Inomata

**Affiliations:** ^1^ Department of Gastroenterological and Pediatric Surgery Oita University Faculty of Medicine Yufu Japan; ^2^ Department of Surgery NTT Medical Center Tokyo Shinagawa‐ku Japan; ^3^ Biostatistics Center Kurume University Fukuoka Japan; ^4^ Department of Digestive and General Surgery University of the Ryukyus Hospital Nishihara Japan; ^5^ Department of Surgery Shimane Prefectural Central Hospital Izumo Japan; ^6^ Department of Gastroenterological, Breast and Endocrine Surgery Yamaguchi University Graduate School of Medicine Ube Japan; ^7^ Department of Digestive and Transplant Surgery Tokushima University Hospital Tokushima Japan; ^8^ Division of Gastrointestinal and Pediatric Surgery, Department of Surgery Tottori University Faculty of Medicine Yonago Japan; ^9^ Department of Gastroenterological Surgery I Graduate School of Medicine, Hokkaido University Sapporo Japan; ^10^ Department of Coloproctology Aizu Medical Center Fukushima Medical University Fukushima Japan; ^11^ Department of Coloproctological Surgery Juntendo University Faculty of Medicine Bunkyo‐ku Japan; ^12^ Department of Colorectal Surgery Dokkyo Medical University Shimotsuga‐gun Japan; ^13^ Division of Surgery, Gastrointestinal Center Yokohama Shin‐Midori General Hospital Yokohama Japan; ^14^ Department of Gastroenterological Surgery Osaka International Cancer Institute Osaka Japan; ^15^ Department of Surgery Tohoku University Graduate School of Medicine Sendai Japan; ^16^ Department of Gastroenterological Surgery Tokai University School of Medicine Shibuya Japan; ^17^ Department of Lower Gastrointestinal Surgery Kitasato University School of Medicine Sagamihara Japan

**Keywords:** body mass index, colon cancer, continuous variable, laparoscopic surgery, obesity paradox

## Abstract

**Background:**

The impact of obesity on colon cancer remains unclear. Very few studies of colon cancer surgery have analyzed body mass index (BMI) as a continuous variable, with no such reports from Japan. This study examined the association between BMI as a continuous variable and short‐ and long‐term outcomes of laparoscopic surgery for obese colon cancer patients.

**Methods:**

Obese (BMI ≥25 kg/m^2^) patients who underwent laparoscopic radical surgery for Stage II/III colon cancer at 46 participating centers from 2009 to 2013 were included. Associations between short‐ and long‐term outcomes and BMI as a continuous variable were analyzed by univariate and multivariate regression models.

**Results:**

Among patients meeting the study criteria, 1036 were examined. BMI as a continuous variable correlated with log‐transformed operative time (regression coefficient: 0.02, 95% confidence interval [CI]: 0.012–0.028, *p* < 0.05) and blood loss (odds ratio: 1.089, 95% CI: 1.032–1.149, *p* < 0.05). There was no association between BMI continuous variables and 3‐year relapse‐free survival (RFS) and overall survival. However, 3‐year RFS was possibly better in patients with BMI ≥28.5 kg/m^2^ versus those with BMI <28.5 kg/m^2^ (hazard ratio: 0.682, 95% CI: 0.462–1.008, *p* = 0.055).

**Conclusions:**

This study showed that BMI as a continuous variable correlated with operative time and blood loss. RFS was possibly better in the severely obese patients (BMI ≥28.5 kg/m^2^), suggesting that the prognosis for highly obese colon cancer patients appears to follow the obesity paradox.

## Graphical abstract

Three‐year relapse‐free survival was possibly better in severe obese patients (BMI ≥28.5 kg/m^2^) who underwent laparoscopic surgery for colon cancer. It suggests that the prognosis of highly obese colorectal cancer patients follow the obesity paradox.

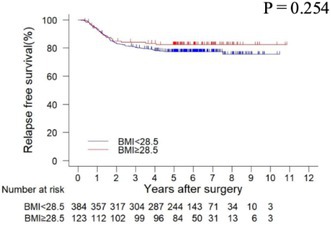

## 1 INTRODUCTION

Laparoscopic surgery has been the standard indication for colorectal cancer for some time, but concrete conclusions on the impact of laparoscopic surgery on obese patients have not been reached. In the JCOG0404 randomized controlled trial^1^ examining survival outcomes of laparoscopic versus open D3 dissection for stage II/III colorectal cancer, the prognosis in the laparoscopic group was significantly worse than that in the open group for the obese patients with colorectal cancer.^2^ However, the number of patients with a body mass index (BMI) ≥30 kg/m^2^ in the JCOG0404 study was small and insufficient to draw conclusions. In contrast, a systematic review examining the benefit of laparoscopic colorectal resection of colorectal cancer in obese patients with BMI ≥30 kg/m^2^ versus nonobese patients with BMI <30 kg/m^2^ found an increased likelihood of postoperative complications in the obese patients, but no difference in oncologic adequacy, suggesting that laparoscopic colorectal resection may be a reasonable treatment for obese patients with colorectal cancer.^3^ This systematic review included only a few cases and an insufficient number of studies to analyze long‐term results. In recent years, some reports^4^, ^5^ have suggested that obese patients have a better prognosis in colorectal cancer, whereas others^6^, ^7^ have suggested that these patients have a worse prognosis. As no concrete conclusion has been reached, our group conducted a large‐scale retrospective multicenter study in Japan. We reported that the long‐term results of laparoscopic surgery for obese colon cancer patients were comparable to those of open surgery but superior in terms of short‐term results.^8^ As a companion study, the present study examined the effect of obesity on the long‐term and short‐term outcomes of laparoscopic surgery in obese patients with colorectal cancer using BMI as a continuous variable.

There are very few such studies reporting the short‐ and long‐term outcomes of laparoscopic surgery for obese colorectal cancer patients using BMI as a continuous variable, and none have been reported in Japan. It should be fully recognized that previous domestic and international reports on the usefulness of laparoscopy for obese patients have not been consistent. In this context, the present study was devised to help provide important scientific evidence for the usefulness of laparoscopy for obese patients by evaluating the degree of BMI in these patients and their short‐ and long‐term outcomes following laparoscopic surgery. Thus, the purpose of this study was to retrospectively examine the short‐ and long‐term outcomes of laparoscopic surgery in obese patients with colorectal cancer by using BMI as a continuous variable.

## 2 MATERIALS AND METHODS

This study was a subgroup analysis of the LOVERY study (UMIN000033529), which retrospectively collected data on laparoscopic and open colorectal cancer surgery for obese patients in Japan. Data for obese patients (BMI ≥25 kg/m^2^) who underwent laparoscopic or open surgery for stage II or III colon cancer between January 2009 and December 2013 were collected by the Japan Society of Laparoscopic Colorectal Surgery from 46 hospitals participating in the laparoscopic versus open surgery for obesity study (the LOVERY study). Patients who had undergone nonradical surgery and those for whom information on tumor location, metastasis, differentiation, and adjuvant chemotherapy were missing were excluded. Finally, patients who had undergone curative surgery for colon cancer and for whom sufficient information was available to investigate their prognosis were included in this study.

In this study, we primarily treated BMI as a continuous explanatory variable and examined its association with the study endpoints. The primary endpoint was the 3‐year relapse‐free survival rate (RFS), which was defined as the duration from the date of the initial surgery to the date of recurrence or death from any cause, whichever occurred first. The secondary endpoints included the 3‐year overall survival rate (OS), bleeding, operation time, extent of central lymph node dissection, number of lymph nodes examined, postoperative complications, 30‐day postoperative mortality, length of hospital stay, and the site of initial recurrence. The 3‐year OS was defined as the duration from the date of the initial surgery to death from any cause. This study followed the Japanese Classification of Colorectal, Appendiceal, and Anal Carcinoma defined by the Japanese Society for Cancer of the Colon and Rectum (JSCCR).^9^ Postoperative complications were classified according to the Clavien–Dindo classification.

### 2.1 Statistical analysis

The primary analysis population was patients scheduled to undergo laparoscopic surgery and in whom the primary endpoint, RFS, was confirmed. All analyses were performed on this population, but as a preplanned supplementary analysis, we also analyzed a population excluding patients who were converted to laparotomy. Continuous variables were summarized using medians, interquartile ranges, and ranges. Categorical variables were summarized using the numbers of cases and percentages. The associations between short‐ and long‐term outcomes and 11 potential risk factors, including BMI as a continuous variable, were analyzed by univariate regression models. Multivariate regression models, which included other factors, were used to analyze outcomes that suggested associations with BMI (*p* < 0.20). This *p*‐value threshold was based on the Akaike Information Criterion (AIC). When selecting a model using AIC, selecting or not selecting one explanatory variable is equivalent to performing a test with a significance level of 0.157.^10^ Based on this, a slightly more lenient value of 0.2 was set as the threshold because of the univariate analysis. Operative time was logarithmically transformed and then fitted to a linear regression model. Logistic regression models were used to analyze complications and postoperative blood loss, binarized with a cutoff value of 60 mL. This cutoff value was set on the basis of the third quartile of blood loss to avoid overly reducing the number of events and to examine the association with factors when blood loss was more significant than usual. RFS and OS were analyzed using Cox proportional hazards models. If relationships between BMI as a continuous variable and outcomes were suggested in the multivariate analyses (*p* < 0.20), BMI was also dichotomized at the third quartile, 28.5 kg/m^2^, and analyzed using multivariate regression models. Regarding this BMI cutoff value, because there were only 131 cases (12.6%) with a BMI of 30 kg/m^2^ or above, which is considered obese according to World Health Organization standards, it was decided in this study to set the cutoff value at the third quartile, 28.5 kg/m^2^, and to define values less than this value as mild obesity and values equal to or higher than this value as severe obesity. Survival curves for RFS in these two BMI groups were estimated by the Kaplan–Meier method. All statistical analyses were performed using R software v.4.1.1 (R Core Team, Vienna, Austria). *p* < 0.05 were considered statistically significant.

## 3 RESULTS

**FIGURE 1:**
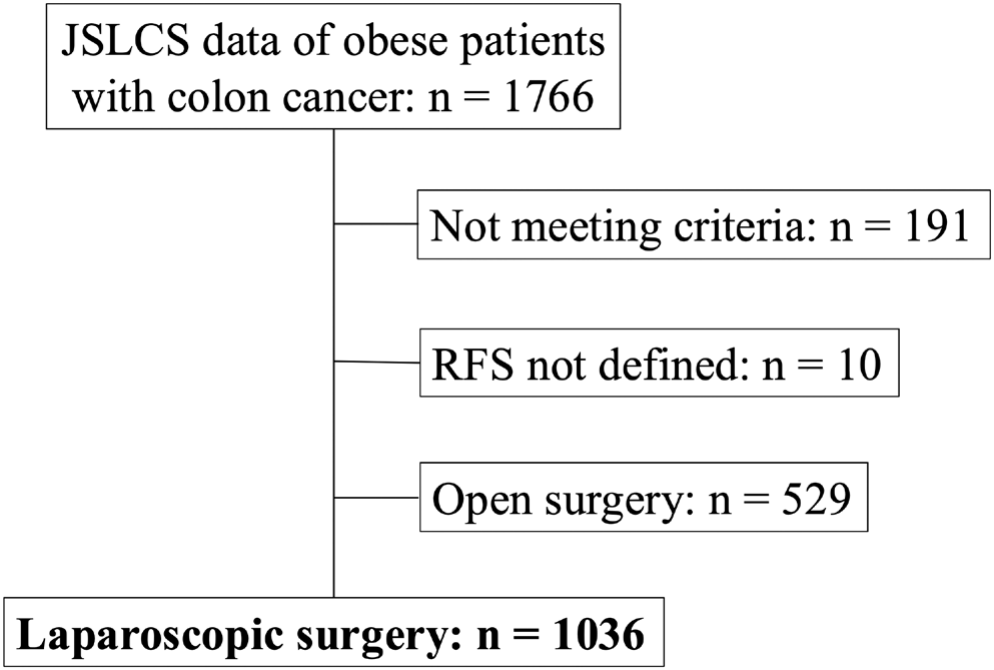
Flowchart for selection of enrolled patients. From a total of 1766 patients, we excluded 191 patients for not meeting the eligibility criteria, 10 patients due to undefined relapse‐free survival, and 529 patients with open surgery, thus leaving 1036 patients with laparoscopic surgery for inclusion in the study. JSLCS, Japan Society of Laparoscopic Colorectal Surgery.

Of the 1766 patients enrolled in the LOVERY study, 191 who did not meet the eligibility criteria were excluded. Among the remaining 1575 patients, 10 patients for whom RFS was not stated and 529 patients who underwent open surgery were excluded, thus leaving 1036 patients with laparoscopic surgery for examination (Figure 1).

The background factors and outcomes in this study are summarized in Tables 1 and 2. The median BMI was 26.8 kg/m^2^, with a range of 25.0–47.9 kg/m^2^. The study included 397 women (38.3%) and 639 men (61.7%) with a median age (range) of 66 (25.0–79.0) years. The study included 513 cases (49.5%) of hypertension, 269 (26.0%) of diabetes mellitus, 55 (5.3%) of cerebrovascular disease, 59 (5.7%) of respiratory disease, and 126 cases (12.2%) of cardiovascular disease. Regarding the T factor, 121 cases (11.7%) were classified as Tis/T1/T2, and 915 cases (88.3%) were classified as T3/T4a/T4b. For the N factor, 536 cases (51.7%) were classified as N0 and 500 cases (48.3%) as N1/N2/N3. Tumor locations were on the right side (cecum, ascending colon, and transverse colon) in 383 cases (37.0%) and on the left side (descending colon, sigmoid colon, and rectum) in 653 cases (63.0%). Adjuvant chemotherapy was administered in 507 cases (48.9%), whereas 528 cases (51.0%) did not receive it, and 1 case was unknown. The median postoperative stay in hospital was 10 (0–739) days, median operative time was 226.5 (80–1340) minutes, median blood loss was 20 (0–2000) mL, and median number of lymph nodes dissected was 19 (1–79). Overall complications of ≥Grade 2 were 113 (10.9%), and those of ≥Grade 3 were 63 (6.1%). Ileus of ≥Grade 2 was observed in 18 patients (1.7%), and that of ≥Grade 3 was observed in eight patients (0.8%). There were 13 (1.3%) wound infections of ≥Grade 2 and 5 (0.5%) of ≥Grade 3.

**TABLE 1:** Summary of background factors.

Factor	Overall	BMI <28.5	BMI ≥28.5
	*N* = 1036^a^	*N* = 782^a^	*N* = 254^a^
BMI, kg/m^2^	26.8 (25.8–28.4) [25.0, 47.9]	26.3 (25.6–27.1) [25.0, 28.4]	30.0 (29.1–31.6) [28.5, 47.9]
Age, years	66 (60–73) [25, 79]	66 (60–73) [31, 79]	65 (59–71) [25, 79]
Gender			
Female	397 (38.3%)	273 (34.9%)	124 (48.8%)
Male	639 (61.7%)	509 (65.1%)	130 (51.2%)
Hypertension			
Yes	513 (49.5%)	375 (48.0%)	138 (54.3%)
No	523 (50.5%)	407 (52.0%)	116 (45.7%)
Diabetes mellitus			
Yes	269 (26.0%)	188 (24.0%)	81 (31.9%)
No	767 (74.0%)	594 (76.0%)	173 (68.1%)
Cerebrovascular disease			
Yes	55 (5.3%)	35 (4.5%)	20 (7.9%)
No	981 (94.7%)	747 (95.5%)	234 (92.1%)
Respiratory disease			
Yes	59 (5.7%)	40 (5.1%)	19 (7.5%)
No	977 (94.3%)	742 (94.9%)	235 (92.5%)
Cardiovascular disease			
Yes	126 (12.2%)	93 (11.9%)	33 (13.0%)
No	910 (87.8%)	689 (88.1%)	221 (87.0%)
T			
Tis/T1/T2	121 (11.7%)	93 (11.9%)	28 (11.0%)
T3/T4a/T4b	915 (88.3%)	689 (88.1%)	226 (89.0%)
N			
N0	536 (51.7)	409 (52.3%)	127 (50.0%)
N1/N2/N3	500 (48.3%)	373 (47.7%)	127 (50.0%)
Location			
Right^b^	383 (37.0)	290 (37.1%)	93 (36.6%)
Left^c^	653 (63.0)	492 (62.9%)	161 (63.4%)
Adjuvant chemotherapy			
Yes	507 (48.9%)	384 (49.1%)	123 (48.4%)
No	528 (51.0%)	397 (50.8%)	131 (51.6%)
N/A	1 (0.1%)	1 (0.1%)	0 (0.0%)

Abbreviations: BMI, body mass index; N/A, not applicable.

^a^ Median (25%–75%) [Range]; *n* (%).

^b^ Cecum, ascending colon, and transverse colon.

^c^ Descending colon, sigmoid colon, rectum (RS, Ra, and Rb).

**TABLE 2:** Summary of outcomes.

Outcome	Overall	BMI <28.5 kg/m^2^	BMI ≥28.5 kg/m^2^
	*N* = 1036^a^	*N* = 782^a^	*N* = 254^a^
Postoperative days in hospital	10 (8–14) [0, 739]	10 (8–14) [0, 739]	10 (8–14) [0, 64]
Operative time, min	227 (182–278) [80, 1340]	220 (180–270) [80, 915]	245 (194–305) [80, 1340]
Blood loss, mL	20 (10–68) [0, 2000]	20 (10–60) [0, 1440]	30 (10–100) [0, 2000]
N/A	2	1	1
Blood loss			
≥20 mL	586 (56.7%)	421 (53.9%)	165 (65.2%)
≥60 mL	294 (28.4%)	200 (25.6%)	94 (37.2%)
Number of lymph nodes dissected	19 (13–27) [1, 79]	19 (13–27) [2, 78]	20 (12–28) [1, 79]
Postoperative complications			
≥Grade 2	113 (10.9%)	86 (11.0%)	27 (10.6%)
≥Grade 3	63 (6.1%)	49 (6.3%)	14 (5.5%)
Anastomotic leak			
≥Grade 2	38 (3.7%)	30 (3.8%)	8 (3.1%)
≥Grade 3	26 (2.5%)	21 (2.7%)	5 (2.0%)
Ileus			
≥Grade 2	18 (1.7%)	12 (1.5%)	6 (2.4%)
≥Grade 3	8 (0.8%)	7 (0.9%)	1 (0.4%)
Wound infections			
≥Grade 2	13 (1.3%)	8 (1.0%)	5 (2.0%)
≥Grade 3	5 (0.5%)	3 (0.4%)	2 (0.8%)

Abbreviations: BMI, body mass index; N/A, not applicable.

^a^ Median (25%–75%) [Range]; *n* (%).

**FIGURE 2:**
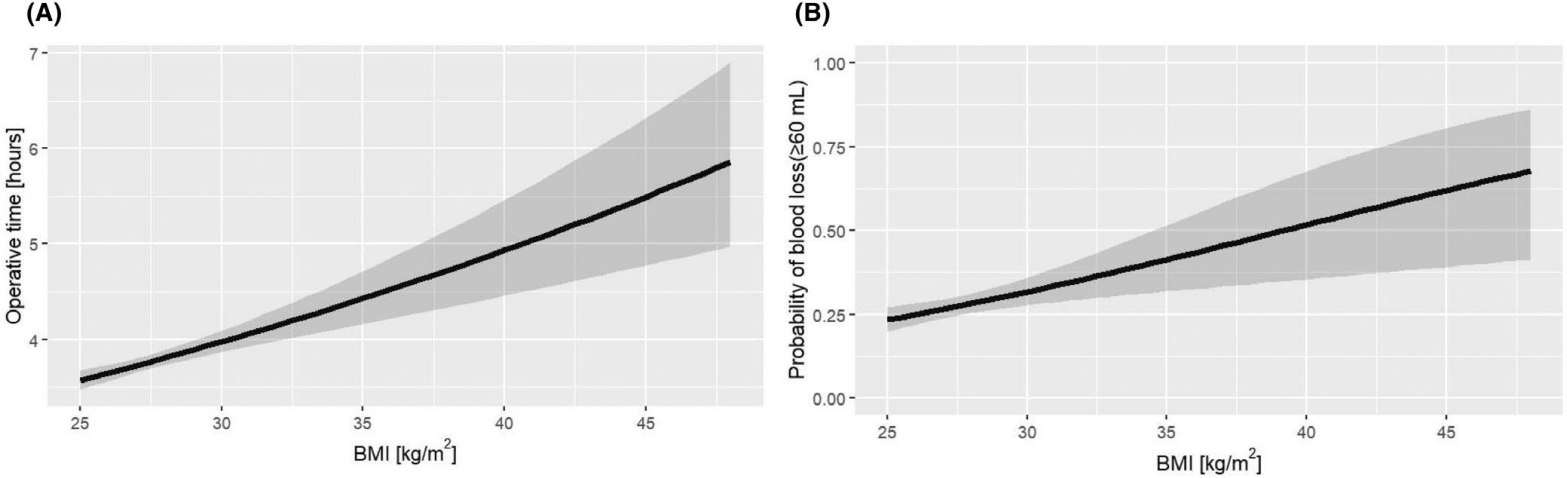
Associations between body mass index (BMI) as a continuous variable and operative time and probability of blood loss. (A) BMI as a continuous variable correlated with operative time (*p* < 0.05). The gray band indicates the range of 95% confidence intervals (CIs). (B) BMI as a continuous variable also correlated with the probability of blood loss (*p* < 0.05). The gray band indicates the range of 95% CIs.

Figure 2A shows the association between BMI as a continuous variable and operative time and indicates that the log‐transformed operative time increased as the BMI value increased (regression coefficient: 0.02, 95% confidence interval [CI]: 0.012–0.028, *p* < 0.05). Figure 2B shows the association between the continuous variable of BMI and the probability of blood loss and also indicates that the probability of blood loss increased as the BMI value increased (OR: 1.089, 95% CI: 1.032–1.149, *p* < 0.05). Table 3 shows the results of the multivariate analysis of the association between the continuous variable of BMI and surgical endpoints. We selected four items that we consider particularly important for short‐term outcomes and complications: overall complications, blood loss, ileus, and anastomotic leakage. We considered blood loss of 60 mL or more to be one of the more important complications. Operative time (regression coefficient: 0.02, 95% CI: 0.012–0.028, *p* < 0.05) and blood loss (≥60 mL, odds ratio [OR]: 1.089, 95% CI: 1.032–1.149, *p* < 0.05) correlated statistically with BMI as a continuous variable. In contrast, ileus (≥G3, OR: 0.747, 95% CI: 0.367–1.150, *p* = 0.255), the number of dissected lymph nodes (*p* = 0.705), overall complications (≥G2, *p* = 0.397; ≥G3, OR: 0.993, 95% CI: 0.888–1.089, *p* = 0.889), and anastomotic leakage (≥G3, OR: 0.939, 95% CI: 0.762–1.096, *p* = 0.497) showed no statistically significant associations.

**TABLE 3:** Association between BMI continuous variables and short‐term outcomes by multivariate analysis.

Outcome	Odds ratio	95% Confidence interval		*p* value
Overall complications (≥Grade 3)	0.993	0.888	1.089	0.889
Blood loss (≥60 mL)	1.089	1.032	1.149	0.002
Ileus (≥Grade 3)	0.747	0.367	1.150	0.255
Anastomotic leakage (≥Grade 3)	0.939	0.762	1.096	0.497

**FIGURE 3:**
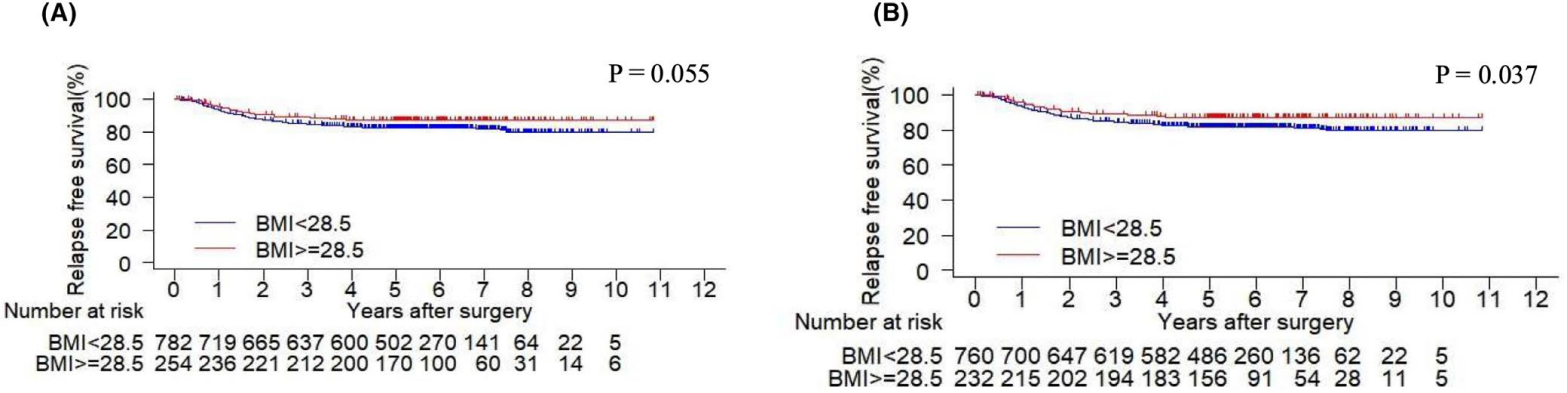
Comparison of Kaplan–Meier curves of relapse‐free survival (RFS) by body mass index (BMI) values of ≥28.5 kg/m^2^ (indicating severe obesity) and <28.5 kg/m^2^ (indicating mild obesity). (A) RFS curve by BMI value shows that 3‐year RFS was possibly better for BMI ≥28.5 kg/m^2^ than for BMI <28.5 kg/m^2^ (OR: 0.682, 95% CI: 0. 462–1.008, *p* = 0.055). (B) RFS curve by BMI value after excluding cases converted to laparotomy shows that 3‐year RFS was significantly better for BMI ≥28.5 kg/m^2^ than for BMI <28.5 kg/m^2^ (OR: 0.644, 95% CI: 0.426–0.973, *p* < 0.05).

**FIGURE 4:**
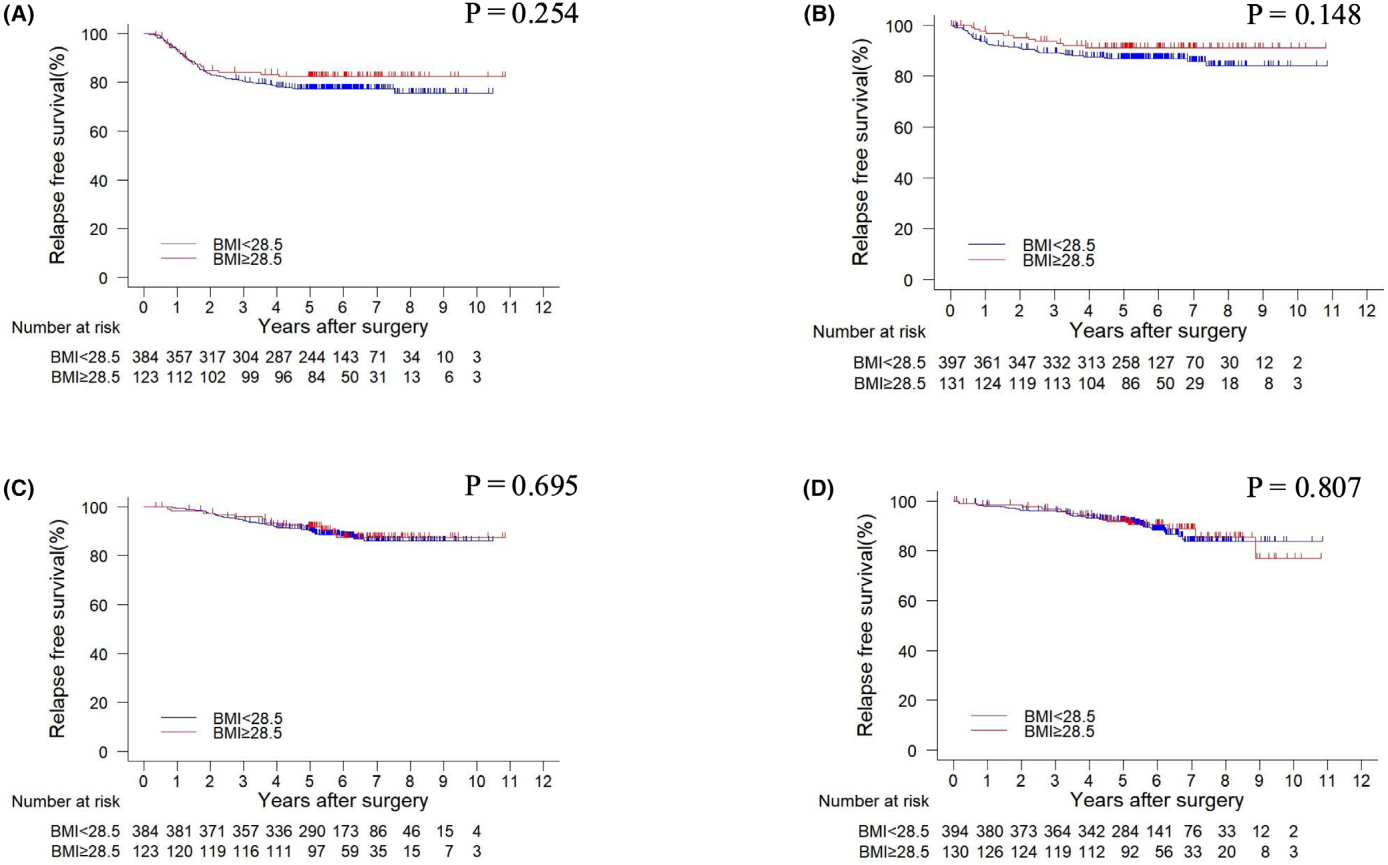
Comparison of Kaplan–Meier curves of relapse‐free survival (RFS) and overall survival (OS) by body mass index (BMI) values of ≥28.5 kg/m^2^ (indicating severe obesity) and <28.5 kg/m^2^ (indicating mild obesity). (A) RFS curves by BMI value in patients with adjuvant chemotherapy. There was no significant difference between BMI ≥28.5 kg/m^2^ and BMI <28.5 kg/m^2^ (HR: 0.758, 95% CI: 0.471–1.220, *p* = 0.254). (B) RFS curves by BMI value in patients without adjuvant chemotherapy. There was no significant difference between BMI ≥28.5 kg/m^2^ and BMI <28.5 kg/m^2^ (HR: 0.618, 95% CI: 0.323–1.185, *p* = 0.148). (C) OS curves by BMI value in patients with adjuvant chemotherapy. There was no significant difference between BMI ≥28.5 kg/m^2^ and BMI <28.5 kg/m^2^ (HR: 0.884, 95% CI: 0.477–1.639, *p* = 0.695). (D) OS curves by BMI value in patients without adjuvant chemotherapy. There was no significant difference between BMI ≥28.5 kg/m^2^ and BMI <28.5 kg/m^2^ (HR: 0.927, 95% CI: 0.507–1.698, *p* = 0.807).

No significant association was found between the continuous variable of BMI and 3‐year RFS (hazard ratio [HR]: 0.948, 95% CI: 0.886–1.015, *p* = 0.127) and 3‐year OS (HR: 0.966, 95% CI: 0.892–1.047, *p* = 0.406) (Tables S2 and S3). Although 3‐year RFS was not statistically significantly better in the patients with severe obesity (BMI ≥28.5 kg/m^2^) compared to those with mild obesity (BMI <28.5 kg/m^2^), it appeared to be possibly better than in those with mild obesity (HR: 0.682, 95% CI: 0.462–1.008, *p* = 0.055) (Figure 3A). However, when the 44 patients with conversion to laparotomy were excluded, 3‐year RFS was significantly better in the patients with severe obesity compared to those with mild obesity (HR: 0.644, 95% CI: 0.426–0.973, *p* = 0.037) (Figure 3B). When postoperative adjuvant chemotherapy was administered, there was no significant difference in 3‐year RFS between the patients with severe obesity and those with mild obesity (HR: 0.758, 95% CI: 0.471–1.220, *p* = 0.254) (Figure 4A). Similarly, in the absence of postoperative adjuvant chemotherapy, no significant difference in 3‐year RFS was observed between the two groups (HR: 0.618, 95% CI: 0.323–1.185, *p* = 0.148) (Figure 4B). Furthermore, when postoperative adjuvant chemotherapy was administered, the 3‐year OS did not significantly differ between the patients with severe and mild obesity (HR: 0.884, 95% CI: 0.477–1.639, *p* = 0.695) (Figure 4C). Likewise, in the absence of postoperative adjuvant chemotherapy, no significant difference in 3‐year OS was found between the two groups (HR: 0.927, 95% CI: 0.507–1.698, *p* = 0.807) (Figure 4D).

## 4 DISCUSSION

This study evaluated the short‐ and long‐term outcomes of 1036 obese patients (BMI ≥25 kg/m^2^) with colon cancer, with BMI considered as a continuous variable. Operative time and blood loss were found to correlate with BMI continuous variables, but no correlation was observed between BMI continuous variables and 3‐year RFS or OS. Three‐year RFS in the patients with severe obesity was not significantly different from that in those with mild obesity, although a favorable trend was shown. Furthermore, excluding patients who underwent conversion to laparotomy, 3‐year RFS was significantly better in the patients with severe obesity compared to those with mild obesity.

Laparoscopic surgery for obese patients with colorectal cancer has been reported to increase operative time, the rate of conversion to laparotomy,^11^, ^12^ and wound infection^13^ compared to open surgery. Yang et al.^14^ used BMI as a continuous variable in their study but did not examine long‐term prognosis. They reported a J‐shaped relationship between BMI and postoperative complications, with the lowest risk observed in patients with a BMI of 25–30 kg/m^2^. However, the likelihood of postoperative complications increased with BMI above 30 kg/m^2^. Hirpara et al.^15^ compared laparoscopic and open surgery for rectal cancer using BMI as a continuous variable but did not report long‐term results. They found that between laparoscopic and open surgery, increasing BMI caused neither a significant change in the overall complication rate (*p* = 0.572) nor a significant difference in operative time (*p* = 0.491). In the present study, BMI continuous variables correlated with operative time and blood loss but not with overall complications. This is a notable finding, indicating that although higher BMI is associated with increased operative time and blood loss, it does not impact short‐term outcomes, thus suggesting that the quality of laparoscopic surgery in Japan remains high, irrespective of the patient's BMI. Conversely, multicenter studies in the United States^16^ and Germany^17^ reported a higher incidence of all complications in the obese patients with colon cancer.

Recent studies have indicated that obese patients have a better prognosis than nonobese patients for various conditions, including colorectal cancer, heart disease, and type 2 diabetes,^18^, ^19^ a phenomenon known as the “obesity paradox.” As with other diseases, the obesity paradox has been observed in colorectal cancer, with obese patients exhibiting a better prognosis than their nonobese counterparts.^4^, ^5^ One possible explanation for the obesity paradox in tumors is that cancer‐induced cachexia may have a more pronounced impact on patients with a lower BMI.^7^ Another potential mechanism involves adipokines secreted by mast cells, particularly adiponectin, which has been shown to exert antitumor effects.^20^ The present study represents the first large‐scale retrospective multicenter analysis in Japan to examine these phenomena.

Reports indicating a worsening prognosis with increasing BMI^6^, ^7^ show that consistent results regarding the obesity paradox remain to be established. In the present study, we evaluated the long‐term outcomes of laparoscopic surgery for colorectal cancer and found no correlation with the continuous variable of BMI and RFS. However, we observed a trend toward better 3‐year RFS in patients with severe obesity compared to those with mild obesity. When excluding the patients who underwent conversion to laparotomy, the 3‐year RFS was statistically significantly better in the patients with severe versus mild obesity. The reason for conversion to laparotomy may have been greater‐than‐expected local tumor progression, which could have affected RFS. However, BMI as a continuous variable correlated with operative time and blood loss. In the future, it will be necessary to investigate optimal treatment methods based on BMI values that improve prognosis while reducing perioperative complications in the treatment of colorectal cancer. Additionally, a meta‐analysis of colorectal cancer without limiting the surgical procedure also demonstrated an obesity paradox.^4^ The results of the present study suggest that laparoscopic surgery may be similarly subject to the obesity paradox.

In the future, it will be necessary to consider optimal treatment methods based on BMI values that improve prognosis while reducing perioperative complications in the treatment of colorectal cancer. In recent years, robotic surgery has expanded into minimally invasive surgery and is becoming the dominant surgical approach, superseding laparoscopic surgery. This emerging surgical technology has the potential to shorten operation time and reduce blood loss for high BMI patients with colon cancer.

This study has several limitations. First, it is a retrospective study. Second, it did not include the full range of BMI values, which may have limited the ability to fully assess the impact of obesity. Third, BMI may not be an appropriate assessment of obesity as it does not distinguish between fat mass and muscle mass. Consequently, individuals with high muscle mass may have a high BMI and be misclassified as obese. Alternative methods, such as measuring visceral fat and musculoskeletal mass using computed tomography, are more accurate. Fourth, BMI was measured only once before surgery. Han and Boyko^21^ reported an inverse correlation between BMI and mortality in patients with type 2 diabetes when comparing BMI assessed once with maximum BMI obtained from historical weight records. Therefore, measuring maximum BMI could provide additional insights.

## 5 CONCLUSION

The BMI of patients undergoing laparoscopic surgery for colon cancer should always be considered because higher BMI increases operative time and blood loss. Although there was no association between BMI as a continuous variable and RFS, RFS was possibly better in the severely obese patients. After exclusion of cases converted to laparotomy, RFS was significantly better in the severely obese patients. This may suggest that the prognosis for highly obese patients with colon cancer would appear to follow the obesity paradox.

## AUTHOR CONTRIBUTIONS

**Takayuki Aiba:** Conceptualization; data curation; formal analysis; investigation; methodology; project administration; validation; visualization; writing – original draft. **Tomonori Akagi:** Conceptualization; data curation; funding acquisition; investigation; methodology; project administration; resources; validation; visualization; writing – review and editing. **Hidefumi Shiroshita:** Conceptualization; writing – review and editing. **Kentaro Nakajima:** Conceptualization; data curation; project administration; resources; validation. **Tetsuji Ohyama:** Conceptualization; data curation; formal analysis; investigation; methodology; software; writing – review and editing. **Tatsuya Kinjo:** Conceptualization; data curation. **Akiyoshi Kanazawa:** Conceptualization; data curation. **Nobuaki Suzuki:** Conceptualization; data curation. **Takuya Tokunaga:** Conceptualization; data curation. **Manabu Yamamoto:** Conceptualization; data curation. **Nobuki Ichikawa:** Conceptualization; data curation. **Shungo Endo:** Conceptualization; data curation. **Yutaka Kojima:** Conceptualization; data curation. **Takatoshi Nakamura:** Conceptualization; data curation. **Shuji Saito:** Conceptualization; data curation. **Yoshinori Kagawa:** Conceptualization; data curation. **Shinobu Ohnuma:** Conceptualization; data curation. **Seiichiro Yamamoto:** Conceptualization; data curation. **Takeshi Naitoh:** Conceptualization; data curation. **Masafumi Inomata:** Conceptualization; data curation; supervision; writing – review and editing.

## FUNDING INFORMATION

The authors declare that no external funding was received for this study.

## CONFLICT OF INTEREST STATEMENT

Masafumi Inomata is an editorial member of the *Annals of Gastroenterological Surgery*. The other authors declare no conflicts of interest for this article.

## ETHICS STATEMENT

Approval of the research protocol by an Institutional Reviewer Board: Patient data were collected following approval by the ethics committee of each participating institution.

Informed Consent: N/A.

Registry and the Registration No. of the study/trial: This study was registered in 2018 under UMIN 000033529.

Animal Studies: N/A.

## Supporting information


Table S1

Table S2

Table S3

## DISCUSSANT

### PROFESSOR YOSHINAGA OKUGAWA

At first, congratulations for receiving this precious award from “Annals of Gastrointestinal Surgery”. Although I feel that your data includes several novel findings using Japanese multicenter cohort dataset, I have two questions.

First, while the authors presented the patient characteristics of their cohort, they did not provide a detailed analysis of the correlation between patient characteristics (such as gender, age, location of the primary tumor, clinicopathological factors, and operative procedures) and BMI as a continuous variable within their cohort. Since these factors are closely related not only to BMI but also to perioperative and oncological outcomes, this information is crucial for the study.

Second, although the authors provided a summary of various pre‐ and perioperative variables analyzed in this cohort, they only presented the uni‐ or multivariate Cox regression or logistic regression analyses using a few selected variables to assess the impact of severe obesity on short‐term or long‐term outcomes. How did the authors choose these factors? If all covariates were used in these analyses, it would be helpful to present all of this data in a table. This information would be valuable for readers to better understand the clinical impact of severe obesity in CRC patients undergoing laparoscopic surgery.

### DR. TAKAYUKI AIBA

Additional relationships between patient characteristics and BMI as a continuous variable are shown in Table S1. Gender was statistically correlated with BMI as a continuous variable (*p* = 0.003). T factor (*p* = 0.35), N factor (*p* = 0.77), and Location (*p* = 0.805) were not correlated with the continuous variable of BMI.

Univariate and multivariate analyses of RFS are shown in Table S2, and univariate analysis of OS in Table S3. In this study, we did not perform multivariate analysis of OS because we planned to perform multivariate analysis when the *p*‐value for BMI was less than 0.2 in univariate analysis.

### PROFESSOR YOSHIHIRO NABEYA

Congratulations on receiving AGSurg Award, and I have enjoyed your paper. However, I have one simple question. Why did you want to use BMI as a continuous variable? Because it is a little bit difficult to understand the results, and I have a feeling that, in order to show obesity paradox clearly, this study should include all patients with all BMI categories. I mean, the subjects should also include patients with BMI <25 kg/m^2^. How do you think about it?

If your study had to be limited to patients with a BMI of 25 kg/m^2^ or greater, how did you determine the BMI cutoff value of 28.5 kg/m^2^?

### DR. TAKAYUKI AIBA

We used BMI as a continuous variable because we believe it allows us to analyze fine variations in the data and model them in detail.

As you indicated, we believe that the impact of BMI can be better evaluated if all BMI ranges are included. We would like to address this issue in the future. We also set the cutoff value for BMI of 28.5 as the third quartile.

## References

[1] Kitano S , Inomata M , Mizusawa J , Katayama H , Watanabe M , Yamamoto S , et al. Survival outcomes following laparoscopic versus open D3 dissection for stage II or III colon cancer (JCOG0404): a phase 3, randomised controlled trial. Lancet Gastroenterol Hepatol. 2017;2(4):261–268. 28404155 10.1016/S2468-1253(16)30207-2

[2] Saito S , Akagi T , Katayama H , Wakabayashi M , Inomata M , Yamamoto S , et al. Identification of patient subgroups with unfavorable long‐term outcomes associated with laparoscopic surgery in a randomized controlled trial comparing open and laparoscopic surgery for colon cancer (Japan Clinical Oncology Group Study JCOG0404). Ann Gastroenterol Surg. 2021;5(6):804–812. 34755012 10.1002/ags3.12475 PMC8560616

[3] Fung A , Trabulsi N , Morris M , Garfinkle R , Saleem A , Wexner SD , et al. Laparoscopic colorectal cancer resections in the obese: a systematic review. Surg Endosc. 2017;31(5):2072–2088. 27778169 10.1007/s00464-016-5209-y

[4] Li Y , Li C , Wu G , Yang W , Wang X , Duan L , et al. The obesity paradox in patients with colorectal cancer: a systematic review and meta‐analysis. Nutr Rev. 2022;80(7):1755–1768. 35182150 10.1093/nutrit/nuac005

[5] Cybulska‐Stopa B , Ługowska I , Wiśniowski R , Domagała‐Haduch M , Rajczykowski M , Piejko K , et al. Overweight is associated with better prognosis in metastatic colorectal cancer patients treated with bevacizumab plus FOLFOX chemotherapy. Contemp Oncol (Pozn). 2020;24(1):34–41. 32514236 10.5114/wo.2020.94728 PMC7265962

[6] Patel GS , Ullah S , Beeke C , Hakendorf P , Padbury R , Price TJ , et al. Association of BMI with overall survival in patients with mCRC who received chemotherapy versus EGFR and VEGF‐targeted therapies. Cancer Med. 2015;4(10):1461–1471. 26211512 10.1002/cam4.490 PMC4618617

[7] Kasi PM , Zafar SY , Grothey A . Is obesity an advantage in patients with colorectal cancer? Expert Rev Gastroenterol Hepatol. 2015;9(11):1339–1342. 26366838 10.1586/17474124.2015.1089170

[8] Nakajima K , Akagi T , Kono Y , Shiroshita H , Ohyama T , Saito S , et al. Laparoscopic versus open colectomy for locally advanced colon cancer in obese patients: a nationwide, multicenter, propensity score‐based analysis of short‐ and long‐term outcomes. Jpn J Clin Oncol. 2024;55:21–28. 10.1093/jjco/hyae127 PMC11708209 39306724

[9] Japanese Society for Cancer of the Colon and Rectum . Japanese classification of colorectal, appendiceal, and anal carcinoma: the 3d English edition [secondary publication]. J Anus Rectum Colon. 2019;3(4):175–195. 31768468 10.23922/jarc.2019-018 PMC6845287

[10] Steyerberg EW . Selection of main effects. Clinical prediction models: a practical approach to development, validation, and updating. Cham: Springer International Publishing; 2019. p. 207–225.

[11] Makino T , Shukla PJ , Rubino F , Milsom JW . The impact of obesity on perioperative outcomes after laparoscopic colorectal resection. Ann Surg. 2012;255(2):228–236. 22190113 10.1097/SLA.0b013e31823dcbf7

[12] Zhou Y , Wu L , Li X , Wu X , Li B . Outcome of laparoscopic colorectal surgery in obese and nonobese patients: a meta‐analysis. Surg Endosc. 2012;26(3):783–789. 22011944 10.1007/s00464-011-1952-2

[13] Miyamoto Y , Ishii T , Tashiro J , Satoh T , Watanabe M , Baba H , et al. Effects of obesity on the outcome of laparoscopic surgery for colorectal cancer. Surg Today. 2014;44(7):1293–1299. 24185897 10.1007/s00595-013-0718-y

[14] Yang PF , Ang ZH , Badiani S , Berney CR , Morgan MJ . Impact of obesity on patients undergoing surgery for rectal cancer in Australia and New Zealand. Int J Colorectal Dis. 2023;38(1):163. 37289290 10.1007/s00384-023-04447-0 PMC10250449

[15] Hirpara DH , O'Rourke C , Azin A , Quereshy FA , Wexner SD , Chadi SA . Impact of BMI on adverse events after laparoscopic and open surgery for rectal cancer. J Gastrointest Cancer. 2022;53(2):370–379. 33660225 10.1007/s12029-021-00612-2

[16] Smith RK , Broach RB , Hedrick TL , Mahmoud NN , Paulson EC . Impact of BMI on postoperative outcomes in patients undergoing proctectomy for rectal cancer: a national surgical quality improvement program analysis. Dis Colon Rectum. 2014;57(6):687–693. 24807592 10.1097/DCR.0000000000000097

[17] Gebauer B , Meyer F , Ptok H , Steinert R , Otto R , Lippert H , et al. Impact of body mass index on early postoperative and long‐term outcome after rectal cancer surgery. Visc Med. 2017;33(5):373–382. 29177167 10.1159/000479852 PMC5697505

[18] Li S , Wang Z , Huang J , Fan J , Du H , Liu L , et al. Systematic review of prognostic roles of body mass index for patients undergoing lung cancer surgery: does the ‘obesity paradox’ really exist? Eur J Cardiothorac Surg. 2017;51(5):817–828. 28040677 10.1093/ejcts/ezw386

[19] Carnethon MR , Rasmussen‐Torvik LJ , Palaniappan L . The obesity paradox in diabetes. Curr Cardiol Rep. 2014;16(2):446. 24408674 10.1007/s11886-013-0446-3

[20] Kim JW , Kim JH , Lee YJ . The role of adipokines in tumor progression and its association with obesity. Biomedicine. 2024;12(1):97. 10.3390/biomedicines12010097 PMC10813163 38255203

[21] Han SJ , Boyko EJ . The evidence for an obesity paradox in type 2 diabetes mellitus. Diabetes Metab J. 2018;42(3):179–187. 29885111 10.4093/dmj.2018.0055 PMC6015958

